# Fruit and vegetable consumption during the COVID-19 lockdown in Sri Lanka: an online survey

**DOI:** 10.1186/s41110-022-00161-z

**Published:** 2022-06-17

**Authors:** Piumika Sooriyaarachchi, Tormalli V. Francis, Ranil Jayawardena

**Affiliations:** 1 School of Exercise & Nutrition Sciences, Faculty of Health, Queensland University of Technology, Brisbane, QLD Australia; 2 Health and Wellness Unit, Faculty of Medicine, University of Colombo, Colombo, Sri Lanka; 3 Department of Physiology, Faculty of Medicine, University of Colombo, Colombo, Sri Lanka

**Keywords:** Fruits and vegetables, Intake, Home-grown, COVID-19, South East Asia

## Abstract

**Purpose:**

The COVID-19 pandemic has drastically altered the dietary patterns of individuals. This study aimed to investigate the changes in the purchase and consumption of fruit and vegetables in Sri Lanka during the COVID-19 pandemic.

**Methods:**

An online cross-sectional survey assessed the self-reported changes in fruit and vegetable consumption and purchase using Google forms. Logistic regression analyses were performed to assess the association between decreased consumption of imported fruits and increased home-grown food intake with socio-demographic variables.

**Results:**

Among the 3621 survey respondents, 63.0% and 43.3% reported a decreased intake of imported and local fruits purchased from the market, respectively. Although the overall vegetable consumption has declined, the leafy vegetable consumption has increased by 40.7%. Imported fruit intake has significantly reduced among youngsters, males, respondents living in municipal areas, employed, and those with lower monthly incomes. Among the respondent, 48.9% declared an increased consumption of home-grown fruits or vegetables. Responders living away from Colombo and rural areas were more likely to report a higher intake of home-grown fruits and vegetables (OR 2.021; 95% CI, 1.762–2.318, *P* < 0.001). Employed males residing in municipal areas were less likely to report an increased intake (OR 0.689; 95% CI, 0.574–0.827, *P* < 0.001).

**Conclusion:**

Purchase of imported and local fruits from the market has reduced. Although the overall vegetable consumption was decreased, there has been an increase in the consumption of leafy vegetables. Furthermore, consumption of home-grown fruits and vegetables has increased considerably. Well-established food distribution programs are essential in future pandemics to promote healthy eating.

## Introduction

Fruit and vegetables are essential food groups with unique health properties. Low intake of fruit and vegetable is a major modifiable risk factor leading to the global rise in chronic disease burden [1]. It is the third most significant dietary risk factor for deaths and disability-adjusted life-years [2]. According to epidemiologic research, increased consumption of fruit and vegetable is linked to a greater life span [3], lower risk of cancers [4], reduced incidence of type 2 diabetes mellitus (T2DM) [5], better cardiovascular health [6], improved mental health [7], and effective weight management [8]. Many nutrients and phytochemicals in fruit and vegetables are associated with a reduced risk for cardiovascular disease (CVD) [9]. Because of their low energy density and glycemic load and high vitamin content, fruit and vegetables help prevent T2DM [10]. Furthermore, including fruit and vegetables in one’s diet helps to prevent obesity by lowering overall energy density and allowing ingestion of satisfying portions while reducing calorie intake [8]. In addition, fruit and vegetables provide antioxidants like vitamin C, beta-carotene, and vitamin E that can reduce inflammation and boost immune function [11].

A healthy immune system is essential in defending against viral infections, including the recent outbreak of the new coronavirus disease (COVID-19). The degree of immunological dysfunction was found to be correlated with the disease severity in COVID-19 [12]. Furthermore, individuals with pre-existing co-morbidities such as cardiovascular disease, hypertension, diabetes, and obesity are at a substantially greater risk of mortality from COVID-19 [13]. Administration of nutrients such as vitamins C, D, E, zinc, and omega-3 fatty acids have demonstrated potential beneficial effects in reducing SARS-CoV-2 viral load and the length of hospitalization [14–16]. Furthermore, it has been reported that the use of vitamin D supplements without overdosing would be a nutritional strategy for the severe reduction of COVID-19 [17, 18]. Therefore, a diet rich in fruits and vegetables which contain anti-inflammatory elements, including carotenoids, vitamin C, and polyphenols, is highly recommended [19].

Generally, almost all South Asian countries appear to consume extremely low quantities of fruit and vegetables, lower than the WHO recommendations. A recent systematic review reported that South Asians consumed between 0.1 and 2.61 fruit servings per day, regardless of gender, with Sri Lanka and Bangladesh reporting the lowest intake (0.43 servings/day) [20]. The daily intake of vegetable servings among South Asians ranged from 0.9 to 4.0, whereas Sri Lankan had beyond three servings per day. A population-based survey conducted in China reported that 41.5% of individuals failed to meet the total fruit and vegetable consumption (≥ 400 g/day) recommended by the WHO [21].

Unfortunately, consuming more fruit and vegetables has become more challenging during the COVID-19 pandemic period. Because of the decreased frequency of grocery shopping, people are consuming less fresh fruit and vegetables and more highly processed meals, including convenience foods, snacks, junk foods, and ready-made cereals that are rich in fats, sugars, and salt [22].

Food insecurity was widespread due to the economic slowdown and job losses caused by the COVID-19 pandemic [23]. Food insecurity may lead to serious public health consequences as it is linked to low diet quality [24]. Food-insecure individuals consume lesser fruit and vegetables, which raises their risk of chronic diseases such as T2DM and cardiovascular disease, as well as mortality [25, 26]. Therefore, low fruit and vegetable intake may have a role in the increased prevalence of chronic diseases linked to food insecurity [27]. Hence, the relationship between food insecurity, nutrition quality, and chronic diseases is of significant concern during the COVID-19 period.

The COVID-19 lockdown has affected the dietary habits and nutritional patterns of people all over the world. Although it is recommended to eat fruit and vegetables to help the immune system, especially during a pandemic, a survey in Kuwait found that more than 70% of respondents did not consume the USDA’s recommended minimum of 5 servings of fruit and vegetables per day during the pandemic [28]. In a national survey among US adults during the mandatory quarantine, 28.2% reported eating less non-starchy vegetables, and 33.4% reported a decreased intake of fruits [29]. Reduced intake could be attributed to a variety of factors, including limited availability of fruit and vegetables and limited food store operating hours due to quarantine [28]. In addition, due to financial constraints, those who are unable to buy larger quantities of food at one time may purchase less-expensive items with long shelf lives [30]. Moreover, panic buying and stocking, supply shortages, export restrictions, closure of borders, or reduced manufacturing capacity have contributed to the lower intake of fruit and vegetables during the lockdown period [31, 32]. Since consumers were worried about the food supply chain collapsing, many people including Sri Lankans started to grow their own fresh fruit and vegetables in their home gardens to face problems with food supply [33, 34].

It is evident that the lockdown period may have compromised opportunities to continue eating the recommended amount of fresh fruit and vegetables. Therefore, this study examines the changes in fruit and vegetable consumption patterns among Sri Lanka residents during the COVID-19 lockdown.

## Methods

### Study design and population

A cross-sectional survey was carried out among Sri Lanka citizens (≥ 16 years) during the COVID-19 lockdown period from the 27th of May to the 2nd of June 2021. The survey was web-based and used Google forms to study the changes in their food habits throughout this period. The online survey was shared through social media platforms, and the participants were recruited voluntarily. The participants gave their informed consent and filled out a self-reporting questionnaire. The detailed methodology of recruiting participants for the web-based survey is published elsewhere [35]. It was necessary to respond to all of the questions in order to submit the questionnaire. Each questionnaire was completed and then uploaded to the Google platform, where the final database was obtained as a Microsoft Excel sheet. Participants were not offered any incentives, and active promotion of the questionnaire was entirely voluntary.

### Inclusion and exclusion criteria

The inclusion criteria were citizens and residents of Sri Lanka age ≥ 16 years old, either male or female. Respondents excluded from the study were those (1) who had illnesses/conditions that can affect their normal dietary patterns, including pregnancy; (2) not living in Sri Lanka; and (3) who did not complete the questionnaire appropriately.

### Sample size calculation

The sample size was calculated using the online Raosoft sample size calculator. Assuming the Sri Lankan population size is nearly 14.4 million (≥ 16 years), the required calculated sample size was 385 with a response rate of 50%, a confidence level of 95%, and a margin of error of 5%. Anticipating 20% with incomplete forms, the final minimal required sample size was 482.

### Questionnaire

The online survey was in all three official languages, Sinhala, Tamil, and English. The questionnaire consisted of two sections with questions regarding (i) demographics and (ii) changes in fruit and vegetable intake during the COVID-19 epidemic. To assess the change in consumption behavior during the lockdown period, fruits and vegetables were categorized into several groups such as local fruits from the market, imported fruits, home-grown fruits, “hill-county” and “low-country” vegetables, home-grown vegetables, and leafy vegetables. The agro-ecological adaptation of Sri Lankan vegetables divides them into two groups: up-country kinds and low-country types [36]. With regard to education levels, the tertiary education group involved people who completed education up to advanced level (A/L). “Degree or above” category comprises respondents with a bachelor’s degree or even higher educational qualifications. Respondents were asked to indicate their change in consumption with respect to the above food groups as “increased,” “decreased,” or “no change.”

### Statistical analysis

The demographic aspects of the study sample were investigated using descriptive statistics. For categorical data, frequency and percentages were used, and for continuous variables, means and standard deviations were used. The associations among dependent (change in imported fruits consumption, change in consumption of home-grown fruit and vegetables) and independent variables were investigated using multinomial logistic regression models for both univariate and multivariate analysis. All predictor variables (age, gender, district, residential area, ethnicity, education level, employment status, and monthly income) were included in the univariate analysis and then adjusted for all in the multivariate analysis. A *P*-value less than 0.05 was considered statistically significant. Considering the analysis, variables “home-grown fruits” and “home-grown vegetables” were combined to make a single variable as “home-grown fruits and/or vegetables.” Educational groups including no schooling, primary, and secondary education also were combined to form a new category as secondary education or below. All odds ratios (ORs) were calculated in relation to a reference group. By comparing the crude and adjusted odds ratios, possible confounding factors were identified. All analyses were made with IBM SPSS statistics version 23.0 software (IBM, Chicago, IL, USA).

## Results

A total of 3621 respondents at the age of 16 years or more were involved in the analysis after removing incomplete and duplicate results. The mean (SD) age of the participants was 32.98 (± 9.79), with females representing the majority (60.0%) (Table 1). The proportion of respondents from the Colombo district was 38.0%, while the rest were from other districts of the country. Rural respondents made up 40.2% of the total, while city and municipal council regions made up 27.2% and 32.6%, respectively. A large portion of participants was Sinhalese (82.1%) and had a degree (70.0%). The majority of the population was employed (69.3%) and had a monthly salary of more than 100,000 LKR (491.61 USD).

**Table 1:** The odds ratios (OR) for consuming imported fruits by socio-demographic variables

Variable	Total		Consumption of imported fruits decreased			
	(*N* = 3621)		Univariate analysis		Multivariate analysis	
	*N*	%	OR (95% CI)	*P*-value	OR (95% CI)	*P*-value
Age (years)						
16–25	803	22.2	1.113 (0.890–1.394)	0.348	0.530 (0.389–0.723)	** < 0.001**
26–30	892	24.6	0.958 (0.771–1.191)	0.700	0.790 (0.629–0.993)	**0.043**
31–35	747	20.6	1.062 (0.846–1.332)	0.604	0.949 (0.750–1.200)	0.662
36–40	489	13.5	0.958 (0.745–1.232)	0.736	0.914 (0.707–1.181)	0.492
> 40*	690	19.1	1		1	
Gender						
Male	1447	40.0	0.730 (0.631–0.844)	** < 0.001**	0.748 (0.642–0.871)	** < 0.001**
Female*	2174	60.0	1		1	
District						
Not Colombo	2246	62.0	1.368 (1.181–1.585)	** < 0.001**	1.156 (0.974–1.373)	0.098
Colombo*	1375	38.0	1		1	
Area of residence						
Municipal council	1181	32.6	0.695 (0.587–0.822)	** < 0.001**	0.815 (0.668–0.994)	**0.044**
City council	984	27.3	0.848 (0.709–1.015)	0.072	0.939 (0.774–1.140)	0.527
Rural*	1456	40.2	1			
Ethnicity						
Sinhala	2974	82.1	1.034 (0.793–1.348)	0.804	0.972 (0.738–1.280)	0.838
Tamil	352	9.7	1.395 (0.979–1.987)	0.065	1.383 (0.964–1.983)	0.078
Moors and others	295	8.1	1		1	
Education level						
Secondary or below	154	4.3	1.443 (0.989–2.105)	0.057	1.179 (0.786–1.768)	0.427
Tertiary	934	25.8	1.423 (1.201–1.687)	** < 0.001**	1.249 (1.035–1.508)	**0.021**
Degree or above*	2533	70.0	1		1	
Employment status						
Employed	2511	69.3	0.680 (0.555–0.833)	** < 0.001**	0.639 (0.469–0.869)	**0.004**
Unemployed	508	14.0	0.970 (0.739–1.275)	0.829	0.804 (0.581–1.112)	0.188
Full time student	602	16.6	1		1	
Monthly family income LKR (USD)						
< 25,000 (122.90)	313	8.6	2.408 (1.762–3.291)	** < 0.001**	2.002 (1.414–2.833)	** < 0.001**
25,000–49,999 (122.90–245.81)	591	16.3	1.639 (1.300–2.066)	** < 0.001**	1.362 (1.053–1.761)	**0.019**
50,000–99,999 (245.81–491.61)	942	26.0	1.340 (1.097–1.635)	**0.004**	1.194 (0.965–1.478)	0.102
100,000–199,999 (491.61–983.22)	876	24.2	1.078 (0.883–1.317)	0.460	1.025 (0.835–1.258)	0.813
> 200,000 (983.22)*	899	24.8	1		1	

The reference category is no change

*OR* odds ratio; *CI* confidence interval; *P* probability value

^*^Reference variable; Bold *p*-values denote statistical significance at the *p*<0.05 level

**Fig. 1:**
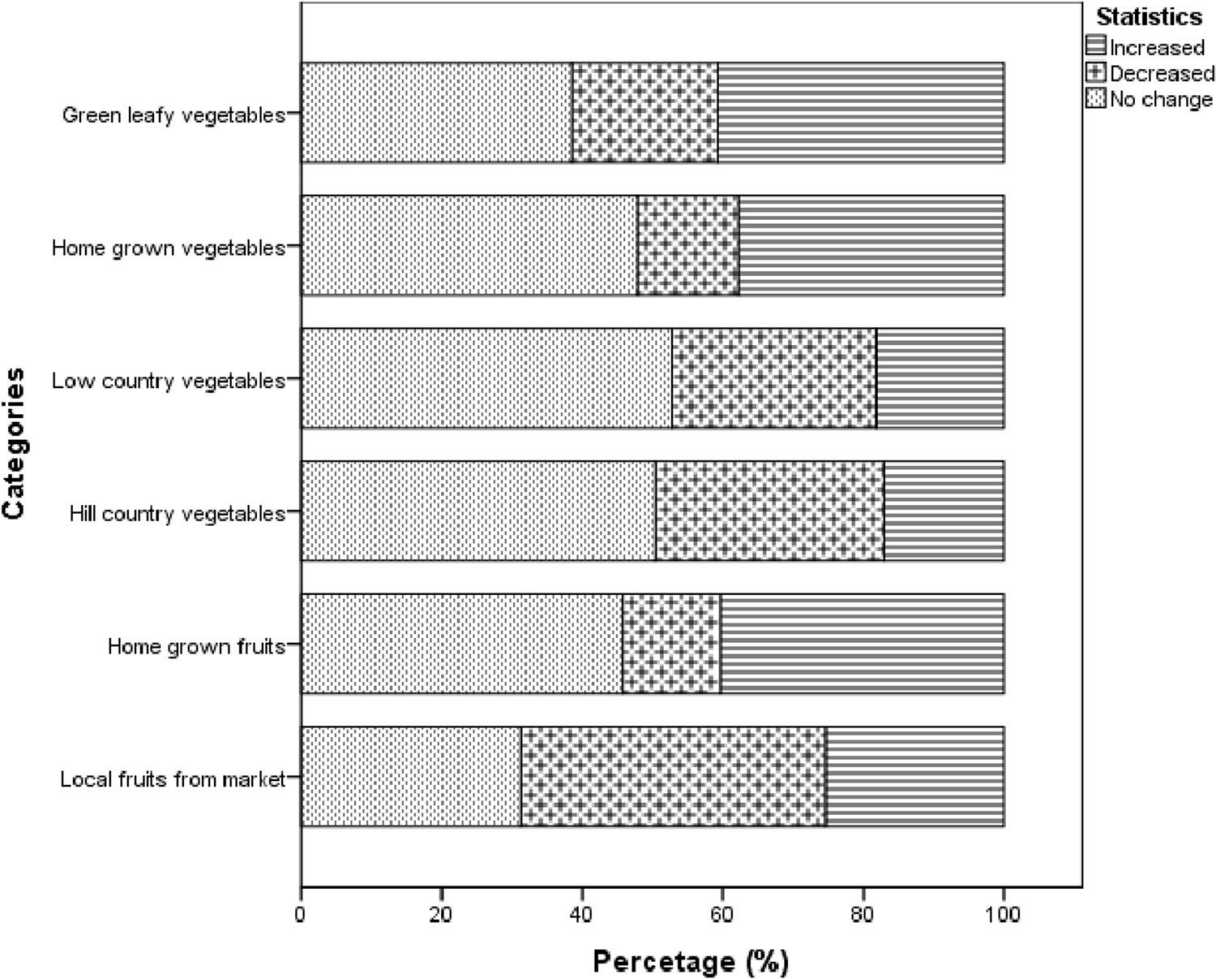
Change in consumption of fruits and vegetables

Changes in the consumption of different fruits and vegetable categories are demonstrated in Fig. 1. In relation to fruit intake, 43.3% of the respondents have decreased the consumption of local fruits bought from the market, while 31.3% and 25.4% reported no change and increased intake, respectively. The survey results showed that 40.4% of respondents had increased their consumption of home-grown fruits. Regarding vegetable consumption, the majority reported no change in low and hill country vegetables intake; however, more participants reported that their intake has decreased than increased. Leafy vegetable consumption increased significantly, with 40.7% of respondents reporting an increased intake. Overall, 47.9% reported no change in intake of home-grown vegetables, while 40.4% and 13.9% informed increased and decreased consumption, respectively.

In relation to consumption of imported fruits, nearly two-thirds (63.0%) of the respondents indicated that their intake has declined, while 30.8% and 6.2% stated that their usage remained stable and has increased, respectively. Demographic predictors of change in intake of imported fruits by univariate and adjusted multiple logistic regression analysis are presented in Table 1. According to the univariate analysis, respondents living out of Colombo were approximately 1.37 (95% CI, 1.181–1.585, *P* < 0.001) times more likely to report a decreased intake than residents in Colombo. Lower monthly income was associated with a higher probability of reporting a reduced intake of imported fruits, with respondents earning less than 25,000LKR (122.90 USD) reporting more than twice the reduction (OR 2.408; 95% CI, 1.762–3.291, *P* < 0.001) as the highest income group.

Moreover, lower education levels also showed higher odds of reporting a reduced intake of imported fruits compared to the highest level. In the adjusted analysis, the age groups of 16–25 and 26–30 years showed significantly lesser odd values for reporting a decreased intake than adults above 40 years of age. Males were significantly less likely to report decreased intake with reference to females (OR 0.748; 95% CI, 0.642–0.871, *P* < 0.001). In particular, the respondents from municipal council areas and who were employed showed significantly lower odd values compared to the rural population and full-time students.

As per the results, 48.9% of respondents have increased their intake of home-grown fruits and/or vegetables, while 51.1% have not. Table 2 presents the unadjusted and adjusted odds ratios of factors associated with the increased intake of home-grown fruits and/or vegetables during the COVID-19 pandemic. The univariate analysis indicated that the chances of reporting an increased intake of home-grown fruits and/or vegetables were significantly associated with respondents’ age. The odds were higher in youngers when compared with people aged 40 years or older. In comparison to respondents who lived in Colombo, those who lived outside of Colombo during the pandemic were twice as likely to report increased consumption of home-grown fruits and/or vegetables. In addition, people with a monthly income of less than 25,000LKR (122.90 USD) had significantly higher odds of consuming home-grown fruits and/or vegetables relative to people in the highest monthly income group (> 200,000LKR/983.22USD). In the multivariable analysis, respondents belonging to Sinhala and Tamil ethnicities were more likely to report an increased intake compared to moors and other ethnicities. Furthermore, when compared to rural areas, people living in the city and municipal council areas were significantly less likely to report a higher intake of home-grown. Employed respondents (OR 0.734; 95% CI, 0.555–0.971, *P* = 0.03) likewise had significantly lesser odds of having more home-grown in comparison to full-time students.

**Table 2:** The odds ratios (OR) for consuming home-grown fruits and vegetables by socio demographic variables

Variable	Consumption of home-grown fruits and vegetables increased			
	Univariate analysis		Multivariate analysis	
	OR (95% CI)	*P*-value	OR (95% CI)	*P*-value
Age (years)				
16–25	1.423 (1.160–1.745)	**0.001**	1.001 (0.752–1.332)	0.996
26–30	1.251 (1.025–1.528)	**0.027**	1.046 (0.846–1.292)	0.678
31–35	1.191 (0.968–1.466)	0.098	1.020 (0.820–1.268)	0.858
36–40	1.023 (0.810–1.291)	0.850	0.951 (0.748–1.209)	0.680
> 40*	1		1	
Gender				
Male	0.783 (0.685–0.894)	** < 0.001**	0.839 (0.728–0.966)	**0.015**
Female*	1		1	
District				
Not Colombo	2.021 (1.762–2.318)	** < 0.001**	1.683 (1.435–1.973)	** < 0.001**
Colombo*	1		1	
Area of residence				
Municipal council	0.508 (0.435–0.593)	** < 0.001**	0.689 (0.574–0.827)	** < 0.001**
City council	0.578 (0.491–0.681)	** < 0.001**	0.699 (0.586–0.834)	** < 0.001**
Rural*	1			
Ethnicity				
Sinhala	2.238 (1.734–2.889)	** < 0.001**	1.970 (1.510–2.569)	** < 0.001**
Tamil	1.722 (1.247–2.377)	**0.001**	1.579 (1.134–2.197)	**0.007**
Moors and others	1		1	
Education level				
Secondary or below	0.974 (0.704–1.349)	0.876	0.722 (0.503–1.035)	0.076
Tertiary	0.942 (0.811–1.095)	0.436	0.795 (0.671–0.942)	**0.008**
Degree or above*	1			
Employment status				
Employed	0.677 (0.566–0.810)	** < 0.001**	0.734 (0.555–0.971)	**0.030**
Unemployed	0.687 (0.542–0.871)	**0.002**	0.762 (0.570–1.018)	0.066
Full time student	1		1	
Monthly family income LKR (USD)				
< 25,000 (122.90)	1.366 (1.055–1.769)	**0.018**	1.010 (0.750–1.359)	0.949
25,000–49,999 (122.90–245.81)	1.058 (0.859–1.302)	0.596	0.819 (0.647–1.037)	0.097
50,000–99,999 (245.81–491.61)	1.139 (0.948–1.367)	0.164	0.947 (0.776–1.155)	0.588
100,000–199,999 (491.61–983.22)	0.950 (0.789–1.145)	0.594	0.893 (0.735–1.084)	0.252
> 200,000 (983.22)*	1		1	

The reference category is no change

*OR* odds ratio; *CI* confidence interval; *P* probability value

^*^Reference variable; Bold* p*-values denote statistical significance at the *p* <0.05 level

## Discussion

To our knowledge, this is the first research study examining the change in the consumption behavior of fruit and vegetables of Sri Lankan residents during the COVID-19 pandemic. Evidence shows that increased consumption of fruit and vegetables benefits the immune system and is also significantly associated with the risk reduction of NCDs [25, 37]. However, it has been revealed that residents of all South Asian countries, including Sri Lanka, appear to consume extremely low amounts of fruit and vegetables, far below than the World Health Organization’s recommended [20]. Therefore, the current research findings are important for developing strategies to improve the consumption of fruit and vegetables during this pandemic.

The findings of our study indicated that the majority of the respondents had a decreased purchasing of local fruits and imported fruits from the market during the pandemic time. This could be due to several reasons. First, the lockdown has impeded vegetable farmers’ access to markets, thus limiting their production and sales capacities. Secondly, in Sri Lanka, some of the regional wholesale economic centers, which are collection and distribution centers of fruits and vegetables, were closed to prevent the virus spread, thus creating massive food waste as well as economic losses to farmers [38]. Thirdly, due to the risk of contracting COVID-19 in stores, customers avoid going to markets and supermarkets [32]. Besides, buying imported products was perceived as particularly risky by consumers due to possible contamination. Moreover, the COVID-19 epidemic resulted in panic buying and an increase in prices of both domestically cultivated and imported fruit and vegetables [32]. Finally, the rising prices could have reduced the purchasing power, particularly among the vulnerable [39].

There was a significant reduction in consumption of imported fruits in people living in Colombo than in other districts of the country. Colombo is the commercial capital city of Sri Lanka with the highest population density and with minimum agriculture [40]. As a result, Colombo city’s food system is likely to be one of Sri Lanka’s most diversified and complicated. As the city is highly reliant on food chains originating in the country’s rural provinces, as well as imported foods, the disruption of food supply chains due to quarantine restrictions has directly influenced the consumption patterns of the respondents in the district of Colombo [41]. The consumption of fruits appeared to reduce with the lower monthly income households of respondents. Similar findings were reported from a study conducted in Kenya and Uganda, where the proportion of food insecure respondents increased by 38% and 44%, respectively, and the regular consumption of fruits decreased by about 30% in both countries during the COVID-19 pandemic when compared to before the pandemic [42].

A large-scale multi-country surveillance program conducted across four South Asian countries, including Sri Lanka, found that inadequate fruit and vegetable intake increased by 10% during the lockdown [43]. According to a research including respondents from four Western countries, daily fruit and vegetable consumption increased from before to throughout the pandemic [44]. Another nationwide survey conducted among Egyptians during the COVID-19 partial lockdown reported a significant increase in the mean weekly consumption of fruit and vegetables (*P* < 0.05) [45]. However, when compared to similar studies carried out in the South Asian region, the current survey’s results showed a similar trend.

Furthermore, it is notable that almost half of the respondents have started consuming home-grown fruit and vegetables during the pandemic. According to survey findings, young people were more likely to consume home-grown fruit and vegetables compared to adults. The closure of schools/universities and working from home may have created more free time for young people and students to engage in home gardening. Furthermore, due to the cancelations of sporting events and the closing of restaurants, clubs, and theaters, youths might be turning to gardening as an outdoor activity. These findings are in line with those of a Canadian survey, which found that 46.2% of new gardeners are between the ages of 24 and 38 years [33]. The same study also reported that majority (52.5%) of respondents who grow fruit and vegetables at home are women, which also agrees with our findings [33]. The nature of the living areas has influenced the practice of home gardening. The availability of more spaces and resources in rural areas of Sri Lanka has made home gardening possible [46].

In addition, home gardens can improve food security, diversity, nutritious value, and the microenvironment around the family home [47]. Home gardening has been shown to effectively reduce depression in persons who were compelled to stay at home during the COVID-19 lockdown period [48]. In addition, home gardening provides easy access to physical activity, which is essential for human health [49]. By promoting home gardening, chemical fertilizer-related disorders can also be reduced [50]. Therefore, the governments need to encourage and increase the awareness of the community about home gardening by offering financial support, tools, and seeds. Also, the citizens should be provided with prior essential knowledge through workshops and distributing resource materials, manuals, and guides [51].

### Study limitations and recommendations

There are several limitations associated with this study. All of the data were self-reported; it is possible that self-reporting bias and a desire to produce socially acceptable responses influenced the results. A web-based survey instrument was employed for its convenience during the COVID-19 lockdown period for data collection, which may have resulted in selection bias. Also, the survey was conducted during a relatively smaller time period. Furthermore, as this study was in a cross-sectional design, no causal associations could be drawn. In addition, we could not collect any quantitative data such as the number of fruit and vegetables servings per day, and also the reasons for change in consumption.

Therefore, future research should focus on determining the reasons for changes in fruit and vegetable consumption and assessing if people meet the daily recommendations. Moreover, planned food distribution programs are essential to a nation during a crisis. Sri Lanka’s food system has already proven to be fragile and inefficient in coping with unprecedented crises. Hence, Sri Lanka requires a well-established and stable central-level mechanism for public food distribution with explicit connections to province and local government entities. Furthermore, a regular monitoring mechanism should be implemented to protect the local food distribution system from probable malpractices and to assure the efficiency of government interventions. In addition, a digital food and nutrition surveillance system with more regular data collection should be set up to monitor vulnerable people in crisis situations.

## Conclusion

The buying of imported and local fruits from the market has decreased during the pandemic. Overall vegetable intake has reduced, although the leafy vegetable consumption has risen. The consumption of home-grown fruit and vegetable intake has also increased, with people living outside of Colombo and in rural regions being more likely to eat home-grown. Our findings are important to take national-level policy decisions on both health and agriculture to improve the consumption of fruit and vegetables during these needy hours.

## Data Availability

The dataset used during and/or analyzed during the current study is available from the corresponding author on reasonable request.
